# Isotope effect on the circular dichroism spectrum of methyl α-D-glucopyranoside in aqueous solution

**DOI:** 10.1038/srep17900

**Published:** 2015-12-14

**Authors:** Yusuke Kanematsu, Yukiko Kamiya, Koichi Matsuo, Kunihiko Gekko, Koichi Kato, Masanori Tachikawa

**Affiliations:** 1 Quantum Chemistry Division, Yokohama City University, Seto 22-2, Kanazawa-ku, Yokohama 236-0027, Japan; 2 Institute of Materials and Systems for Sustainability, Nagoya University, Furo-cho, Chikusa-ku, Nagoya 464-8603, Japan; 3 Hiroshima Synchrotron Radiation Center, Hiroshima University, Higashi-Hiroshima 739-0046, Japan; 4 Institute for Molecular Science and Okazaki Institute for Integrative Bioscience, National Institutes of Natural Sciences, 5-1 Higashiyama Myodaiji, Okazaki, Aichi 444-8787, Japan

## Abstract

H/D isotope effect on the circular dichroism spectrum of methyl α-D-glucopyranoside in aqueous solution has been analyzed by multicomponent density functional theory calculations using the polarizable continuum model. By comparing the computational spectra with the corresponding experimental spectrum obtained with a vacuum-ultraviolet circular dichroism spectrophotometer, it was demonstrated that the isotope effect provides insights not only into the isotopic difference of the intramolecular interactions of the solutes, but also into that of the solute–solvent intermolecular interaction.

Circular dichroism (CD) is one of the most utilized spectroscopic properties for the assignment of the biomolecule structure. Following the recent development of spectroscopic and computational techniques, the combination of measurement and the computation of CD spectra has become an increasingly important approach for the detailed elucidation of molecular structure. Beside proteins and nucleic acids, CD has recently been applied for structural analysis of carbohydrates using vacuum-ultraviolet circular dichroism (VUVCD) spectrophotometer^1^,^2^,^3^,^4^,^5^. For example, Matsuo *et al.* compared the experimental CD spectrum of methyl α-D-glucopyranoside (methyl α-D-Glc, Fig. 1) in aqueous solution with the spectrum obtained by quantum mechanical (QM) calculations based on time-dependent density-functional theory (TD-DFT) with the polarizable continuum model (PCM)^6^,^7^ for the solvent effect and molecular dynamics (MD) simulation for conformational sampling^3^. Their pioneering analysis demonstrated that the static computation of CD spectrum for three representative equilibrium geometries resulted in a substantial difference between the experimental and computational spectra that was then successfully reduced by the use of broad conformational sampling for the accumulation of the spectrum. Matsuo *et al.* revealed that the fluctuation of the intramolecular hydrogen bond orientation strongly influenced the methyl α-D-Glc CD spectrum.

As seen in the above case, the fluctuation of hydrogen atom in hydrogen bonding systems can have a dominant effect on the molecular properties of hydrogen-bonded systems. Such systems are often associated with a significant change in properties on deuteration, *i.e.,* the H/D isotope effect that arises from the isotopic difference of a quantum mechanical nature^8^. The isotope effect is known to slightly modulate the molecular geometry of hydrogen bonded systems, and it sometimes results in a drastic change of the phase-transition temperature^9^, chemical reaction rate^10^, and the nuclear magnetic resonance and infrared absorption spectra^11^. These isotopic difference effects can be used to obtain further detailed information on the behavior of molecules^11^. Although scarcely explored so far, a significant isotope effect can be expected for the CD spectrum of methyl α-D-Glc because of its dependence on the conformational fluctuations shown by Matsuo *et al.*

The H/D isotope effect cannot be predicted by the simple application of the conventional electronic structure calculation and classical MD simulation that ignore the quantum nature of the hydrogen nuclei. As an alternative choice for computing the molecular spectroscopic properties that efficiently considers the significant fluctuation of the hydrogen nuclei in hydrogen bonds and the concomitant H/D isotope effect, Tachikawa *et al.* have developed a multicomponent (MC) scheme for the feasible extension of the QM calculation^12^. Quantum mechanical calculations using this scheme (MC_QM) incorporate the quantum deviation of nuclei from equilibrium geometry, *i.e.,* the nuclear quantum effect, into the molecular property calculation with only slight additional cost^13^. This approach has been recently extended to the combination with the PCM [MC_QM/PCM] for the analysis of the condensed phase^14^.

## Results and Discussion

**Figure 2:**
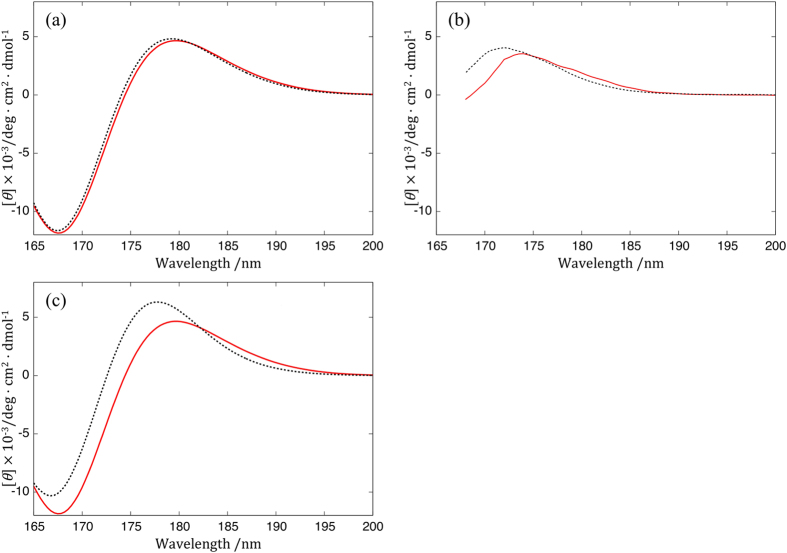
CD spectra of the methyl α-D-Glc (C_7_H_14_O_6_, red) in H_2_O and its isotopologue (C_7_H_10_D_4_O_6_, dashed black) in D_2_O of (a) initial computational prediction, (b) experimental measurement, and (c) revised computation with the scaling of the solvation radius by 1.04.

We have applied the MC_QM/PCM approach for the calculation of the CD spectrum of methyl α-D-Glc in aqueous solution as a tentative probe into the isotope effect on CD, with the calculated spectra shown in Fig. 2(a). We focused on methyl α-D-Glc (C_7_H_14_O_6_) and its isotopologue (C_7_H_10_D_4_O_6_) with deuterated hydroxyl groups. The shape of the obtained spectra agreed with that of the previous study^3^, demonstrating that the present approach is as reasonable as the combination of MD sampling and conventional QM calculation for modeling the fluctuating methyl α-D-Glc in aqueous solution. The isotopic difference of the spectra in Fig. 2(a) shows that the deuteration of the solute will lead to only a tiny shift. Thus, we predict that H/D isotope effect on the CD spectrum can be marginally detectable.

To verify the computational prediction of the isotope effect, we measured the CD spectra of the isotopologues of methyl α-D-Glc by using a vacuum-ultraviolet circular dichroism (VUVCD) spectrophotometer^1^,^2^,^3^. Deuteration was introduced by the substitution of light water solvent with heavy one. Fig. 2(b) shows the observed CD spectra of methyl α-D-Glc in H_2_O and D_2_O. Contrary to computational prediction, a significant blue shift and an increase in the peak intensity were observed on deuteration.

What is the origin of the discrepancy between the predicted and the observed isotope shifts? To answer this question we focused on the isotopic difference of the solute-solvent interaction that was not considered in the above calculation. It is well known that the deuteration of hydrogen-bonding clusters involves the elongation of hydrogen-bonding distances; this is the so-called Ubbelohde effect^11^. Similar isotope effects have also been reported for liquid water, in which the length of the hydrogen-bond in light water was evaluated to be ~4% shorter than that in heavy water^15^. It can be expected that the interaction distances between the hydrophilic solutes and solvent water molecules undergo similar changes with isotopic substitution.

Therefore, we recalculated the CD of methyl α-D-Glc in D_2_O considering the isotope effect on the intermolecular interaction with the deuteration of the solute hydroxyl groups. To describe the elongation of the intermolecular distance on deuteration and the corresponding slight weakening of the solute-solvent interaction, the solvation radius used in PCM was scaled by factors of around 1.04 for D_2_O and alienated from the solute. The results of these calculations are shown in Fig. 2(c) and Supplementary Figure S1. The relevant isotopic difference of the OH-bond length, cavity volume, and the solvation energy are shown in Supplementary Table. We can see that the computational isotope effect in Fig. 2(c) was significantly enhanced to achieve better agreement with the experimental isotope shift shown in Fig. 2(b), which was brought by only a slight alienation of the solvation surface and the relevant change of the geometry and the energy. This result indicates that the obtained isotopic shift strongly depends on the isotopic difference of the solute-solvent interaction. Consequently, the isotope effect on the CD spectrum can offer insight into not only the solute conformations but also the carbohydrate hydration. Such insight is not available by other spectroscopic techniques such as IR and NMR.

## Summary

To summarize, we have analyzed the isotope effect on the CD spectrum of methyl α-D-Glc in aqueous solution experimentally and theoretically. We have observed significant isotopic differences for the peak position and the intensity in spectra. The modification of the solvation surface was essential for reproducing the observed isotope effect on the CD spectrum by MC_QM/PCM calculation, indicating that the isotopic differences are strongly dependent on the solute-solvent interaction. The present results suggest that the isotope effect on CD spectra carries the information about the conformation of the hydroxyl groups and water molecules along the solvation surface; this will provide new insights into the activity of biomolecules including saccharides.

## Method

### VUVCD Measurement

Methyl α-D-Glc of high purity (>98%) was purchased from Sigma-Aldrich (St. Louis, MO) and used without further purification. The sample solutions were freshly prepared by dissolving in H_2_O or D_2_O at a concentration of 10.0 (w/v%). The obtained sample solutions were incubated at room temperature for 1 day prior to performing VUVCD measurements.

The VUVCD spectra of methyl α-D-Glc in H_2_O or D_2_O were measured in the 168–210 nm wavelength range at 25 °C using a VUVCD spectrophotometer at the Hiroshima Synchrotron Radiation Center. A detailed description of the spectrophotometer optical devices is available elsewhere^16^,^17^. The VUVCD measurement was performed using an assembled-type optical cell with CaF_2_ windows^17^. The path length of the cell was adjusted with a Teflon spacer to 50 μm for the measurements from 200 to 180 nm, and to reduce the effects of light absorption by the solvent, no spacer was used for the measurements below 180 nm. The spectra obtained without the spacer were calibrated by normalizing the ellipticities to the spectra measured using a 50 μm spacer in the overlapping wavelength region from 180 to 200 nm. The spectrum was recorded with a 1.0 mm slit, an 8 s time constant, and an 8 nm/min scan speed and by using four accumulations. All spectra were smoothed with the Savitsky–Golay filter. The molar ellipticity [*θ*] was calculated using the molecular weight of the solute. The ellipticity was reproducible within an error of ±5%, which can be mainly attributed to noise and the inaccuracy in the light path length.

### Conformational search

We have performed conformational search using CONFLEX^18^,^19^. After 457 precursory conformers were obtained by CONFLEX with MMFF94s force field and the GB/SA solvation model, 180 unique conformers optimized by CAM-B3LYP/6-31G(d) with the SMD variant of the PCM solvation model^20^ were obtained from the precursors. The obtained structures included 71 GG, 61 GT, and 48 TG hydroxymethyl rotamers of methyl α-D-Glc. TD-CAM-B3LYP/6-311++G(2d,2p)/SMD calculation was applied to obtain the rotatory strength of 40 GG, 40 GT, and 25 TG relatively stable conformers according to the relative energies evaluated by CAM-B3LYP/6-311++G(2d,2p)/SMD calculations for the conformations. All quantum mechanical calculations were performed with the Gaussian 09 package^21^ in which we have implemented the multicomponent scheme calculation.

### Computational CD spectrum of representative GG and GT hydroxymethyl rotamers

We performed the geometry optimization and rotatory strength calculation of methyl α-D-Glc conformers using CAM-B3LYP with the multicomponent scheme (MC_CAM-B3LYP). In MC_CAM-B3LYP calculations, all hydrogen nuclei were treated quantum mechanically. Two isotopologues were investigated; the first isotopologue had only protons as the hydrogen nuclei and four OH groups of the molecule were replaced with the deuterons for the other isotopologue. We used 6-31G(d) electronic basis set for geometry optimization and 6-311++G(2d,2p) basis set for rotatory strength calculations. For both types of calculations, [1s] GTF calculations were used with 24.1825 a.u. exponent value for the proton and 35.6214 a.u. for deuteron optimized for the quantum treatment of hydrogen nuclei in the MC_HF scheme^22^. Electronic excitation calculation based on TD-DFT with CAM-B3LYP functional was performed to obtain the rotatory strength of the optimized geometry, where the occupied and virtual Kohn–Sham orbitals were evaluated with the multicomponent scheme and the excitation of the hydrogen nuclei was neglected. 30 rotatory strengths were calculated for each conformer to construct the CD spectrum according to the equation:


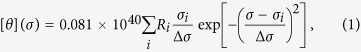


where 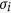 is the excitation energy, 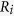 is the corresponding electronic rotatory strength, and 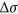 is the bandwidth of each signal that was set to 0.30 eV.

Average CD spectrum among the rotamers GG, GT, and TG hydroxymethyl rotamers have four OH groups that can rotate to give rise to different hydroxyl rotamers. To consider rotation, average CD spectra were constructed on the basis of the Boltzmann distribution of each OH rotamer according to the equation:


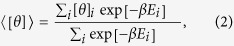


where *E*_*i*_s are the relative energies without the entropic contributions and the temperature in *β* is set to 298 K. The relative populations of GG, GT, and TG hydroxymethyl rotamers in the above averaging were [GG:GT:TG = 0.45:0.45:0.10], which is in reasonable agreement with the previously reported population of [GG:GT:TG = 0.48:0.48:0.04]^23^.

## Additional Information

**How to cite this article**: Kanematsu, Y. *et al.* Isotope effect on the circular dichroism spectrum of methyl α-D-glucopyranoside in aqueous solution. *Sci. Rep.* **5**, 17900; doi: 10.1038/srep17900 (2015).

## Supplementary Material

Supplementary Information

## Figures and Tables

**Figure 1:**
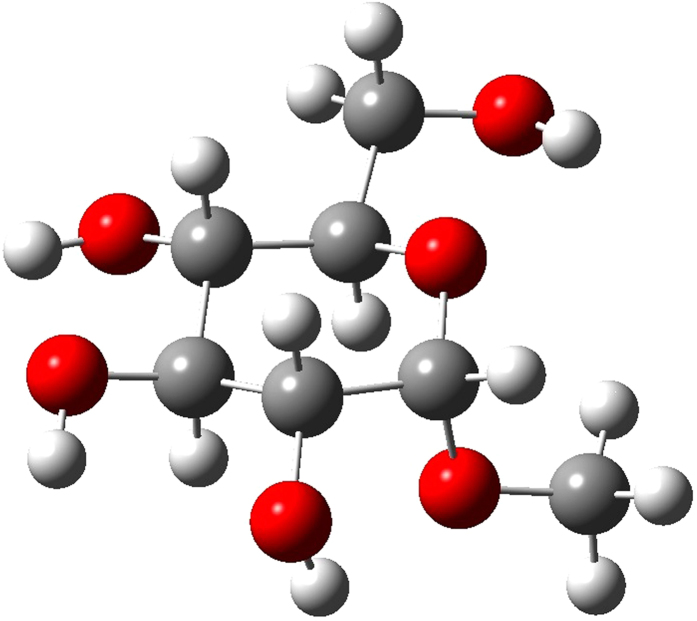
Methyl-α-D-glucopyranoside.
