# Supplementary material for: Isotope effect on the circular dichroism spectrum of methyl α-D-glucopyranoside in aqueous solution
Source: Sci Rep. 2015 Dec 14;5:17900. doi: 10.1038/srep17900 (PMC4677324; doi:10.1038/srep17900)

Supplementary information

# Isotope effect on the circular dichroism spectrum of methyl α-D-glucopyranoside in aqueous solution.

Yusuke Kanematsu[a], Yukiko Kamiya[b], Koichi Matsuo[c], Kunihiko Gekko[c], Koichi Kato[d*], and Masanori Tachikawa[a*]

[a] Quantum Chemistry Division, Yokohama City University, Seto 22-2, Kanazawa-ku, Yokohama 236-0027, Japan

[b] Institute of Materials and Systems for Sustainability, Nagoya University, Furo-cho, Chikusa-ku, Nagoya 464-8603, Japan

[c] Hiroshima Synchrotron Radiation Center, Hiroshima University, Higashi-Hiroshima 739-0046, Japan

[d] Institute for Molecular Science and Okazaki Institute for Integrative Bioscience, National Institutes of Natural Sciences, 5-1 Higashiyama Myodaiji, Okazaki, Aichi 444-8787, Japan.

*e*-mail: tachi@yokohama-cu.ac.jp, kkato@phar.nagoya-cu.ac.jp

S1. Isotopic differences in the geometry and the solvation energy.

The averaged geometrical parameters and the solvation energy obtained by our calculation are summarized in Table S1. Comparing the top and the middle lines of Table S1, we can see the H/D isotopic substitution involved the decrement of the OH-bond length by 0.7%, and solvation energy by 2% (0.5 kcal/mol) on average. The shortening of the OH-bond length upon the deuteration could result in the suppression of the polarization of the bond as "$O^{\delta-}$–$H^{\delta+}$", which would decrease the solute–solvent electrostatic interaction energy. It can also be seen that 4 % extension of the solvation radius involved the increment of the solvation volume by 7%, and the decrement of the solvation energy by 15% (4 kcal/mol). The alienation of the $D_2O$ solvation surface had therefore larger perturbation on the solvation energy than the H/D substitution of OH groups of the solute in our computation.

S2. Sensitivity of the computational CD spectra on the solvation radius.

To investigate the sensitivity of the computational CD spectra to the change of the solvation surface, we calculated the CD spectra with several scaling factors of the

solvation radius. The results are shown in Figure S1, indicating that the scaling factors less than 1.03 brought little change on the shape of the spectra. By contrast, the scaling factors higher than 1.04 involved significant change, which accentuated the H/D isotopic difference of the spectra. The H/D isotopic difference was robust for the scaling factor from 1.04 to 1.05. Given that the solvation energy monotonically decreases as the solvation surface alienates, it can be considered that the energetic difference of solute–solvent interaction by 4 kcal/mol (seen in Table S1) or higher would be required to describe such substantial H/D isotope effect as that experimentally observed on CD spectrum of methyl α-D-glucopyranoside.

Reference

21. Gaussian 09, Revision C01, M.J. Frisch, G.W. Trucks, H.B. Schlegel, G.E. Scuseria, M.A. Robb, J.R. Cheeseman, G. Scalmani, V. Barone, B. Mennucci, G.A. Petersson, H. Nakatsuji, M. Caricato, X. Li, H.P. Hratchian, A.F. Izmaylov, J. Bloino, G. Zheng, J.L. Sonnenberg, M. Hada, M. Ehara, K. Toyota, R. Fukuda, J. Hasegawa, M. Ishida, T. Nakajima, Y. Honda, O. Kitao, H. Nakai, T. Vreven, J. Montgomery, J. A., J.E. Peralta, F. Ogliaro, M. Bearpark, J.J. Heyd, E. Brothers, K.N. Kudin, V.N. Staroverov, R. Kobayashi, J. Normand, K. Raghavachari, A. Rendell, J.C. Burant, S.S. Iyengar, J. Tomasi, M. Cossi, N. Rega, M.J. Millam, M. Klene, J.E. Knox, J.B. Cross, V. Bakken, C. Adamo, J. Jaramillo, R. Gomperts, R.E. Stratmann, O. Yazyev, A.J. Austin, R. Cammi, C. Pomelli, J.W. Ochterski, R.L. Martin, K. Morokuma, V.G. Zakrzewski,

G.A. Voth, P. Salvador, J.J. Dannenberg, S. Dapprich, A.D. Daniels, Ö. Farkas, J.B. Foresman, J. V. Ortiz, J. Cioslowski, and D.J. Fox, (2009).

Table S1. The computational OH-bond length ($r_{OH}$ in Å), the volume of the solvation cavity ($V_{solv}$ in Å$^3$), and the solvation energy ($\Delta G_{solv}$ in kcal/mol) averaged for the 105 isomers of H/D isotopologues of methyl α-D-glucopyranoside with/without the scaling of solvation radius for PCM.

| | Scaling factor | $r_{OH}$ | $V_{solv}$ | $\Delta G_{solv}$ |
|---|---|---|---|---|
| H | 1.00 | 0.993 | 175 | 25.2 |
| D | 1.00 | 0.986 | 175 | 24.7 |
| D | 1.04 | 0.986 | 187 | 21.1 |

Figure S1. Computational CD spectra of H/D isotopologues of methyl α-D-glucopyranoside with the scaling factors from 1.00 to 1.05.

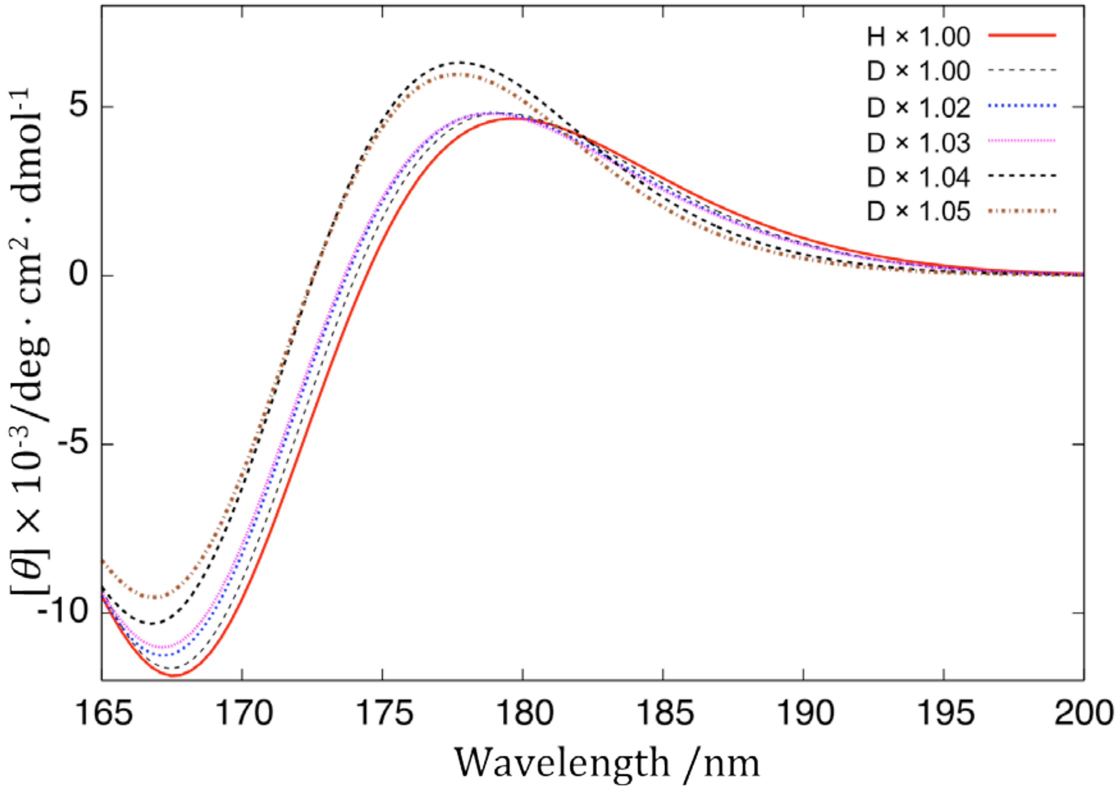

Supplement: Supplementary Information [file srep17900-s1.pdf]
